# Antioxidant Activity and Cardioprotective Effect of a Nonalcoholic Extract of *Vaccinium meridionale* Swartz during Ischemia-Reperfusion in Rats

**DOI:** 10.1155/2013/516727

**Published:** 2013-02-06

**Authors:** Yasmin E. Lopera, Juliana Fantinelli, Luisa F. González Arbeláez, Benjamín Rojano, José Luis Ríos, Guillermo Schinella, Susana Mosca

**Affiliations:** ^1^Laboratorio de Ciencia de Alimentos, Universidad Nacional de Colombia, Sede Medellín, Medellín, Colombia; ^2^CONICET, Centro de Investigaciones Cardiovasculares, Facultad de Ciencias Médicas, Universidad Nacional de La Plata, La Plata, Argentina; ^3^Departament de Farmacologia, Facultat de Farmàcia, Universitat de València, Burjassot, 46100 Valencia, Spain; ^4^Cátedra de Farmacología Básica, Facultad de Ciencias Médicas, Universidad Nacional de La Plata and CIC Provincia de Buenos Aires, La Plata, Argentina

## Abstract

Our objective was to assess the antioxidant properties and the effects against the reperfusion injury of a nonalcoholic extract obtained by fermentation from the Colombian blueberry, mortiño (*Vaccinium meridionale* Swartz, Ericaceae). Antioxidant properties were assessed by *in vitro* systems. To examine the postischemic myocardial function, isolated rat hearts were treated 10 min before ischemia and during the first 10 min of reperfusion with the extract. To analyze the participation of nitric oxide (NO), other experiments were performed in the presence of nitric oxide synthase (NOS) inhibition with N^G^-nitro-L-arginine methyl ester (L-NAME). In cardiac tissue thiobarbituric acid reactive substances (TBARS) concentration, reduced glutathione (GSH) content, endothelial NOS (eNOS), and Akt expression were also measured. The blueberry extract showed higher total phenols and anthocyanins contents, scavenging activity of superoxide radical and systolic and diastolic function was improved, TBARS diminished, GSH was partially preserved, and both NOS and Akt expression increased in hearts treated with the extract. These beneficial effects were lost when eNOS was inhibited. In resume, these data show that the increase of eNOS expression via Akt and the scavenging activity contribute to the cardioprotection afforded by acute treatment with Colombian blueberry extract against ischemia and reperfusion injury.

## 1. Introduction

Oxidative stress is defined as the imbalance between the generation of reactive oxygen species (ROS) and antioxidant defense mechanisms. Myocardial damage induced by ischemia-reperfusion of the heart has been proposed to be caused, at least in part, by ROS production [1–3]. Indirect evidence supporting this hypothesis has been the cardioprotective effects of ROS scavengers and agents capable of inducing antioxidants such as glutathione peroxidase (GSHpx) and superoxide dismutase (SOD) and of supplementing antioxidants [4]. The detrimental action of ROS has naturally led to increased interest in antioxidants, in particular, plant diet-derived antioxidants, as possible therapeutic agents [5, 6]. Unfortunately, the attempts to use synthetic antioxidants to block or attenuate the harmful effects of ROS have produced mixed and mostly negative results [7, 8]. There are various elements involved in this “antioxidant paradox,” such as the selection of dose, degree of collateral blood flow, duration of ischemia, timing of drug administration, and drug delivery method [9]. Reduced nitric oxide (NO) bioavailability is other important factor mediating ischemia and reperfusion injury [10]. Thus, the treatment with NO donors is able to prevent reperfusion-induced cell death [11]. Data from our group [12] and other laboratories [13] show that NO mediates the beneficial effects of natural antioxidants.

The polyphenols present in fruits, green tea, “yerba mate,” black chocolate, or red wine exert antioxidant properties through various mechanisms of action including the ROS scavenging and ROS generation inhibition, thereby preventing damage to lipids, proteins, nucleic acids, and eventually cell damage and death [14]. These properties appear involved in the beneficial effects of those natural products on health previously reported by us [12, 15–17] and other researchers [18, 19]. The genus *Vaccinium* provides a group of plants with promising antioxidant properties. It includes about 400 species, which grow principally in the hillside of tropical mountains. One of them, agraz or Colombian blueberry, is the species *Vaccinium meridionale* Swartz (Ericaceae) which grows in the Andean region of South America at 2300–3300 m and it is a wild bush of 1–4 m high, sometimes until 8 m. The fruits are edible dark purple berries [20]. It is currently available only in the wild and their spontaneous populations are found across the Colombian mountains [21]. Blueberries contain phenolics such as anthocyanins and flavonoids and appear to have the highest antioxidant capacity among fruits and vegetables [22, 23], and the fermented juice, such as in the case of wine, improves its processing and storage effects on anthocyanin composition, color, and appearance [24]. Thus, consumption of berries or their related products could be of importance not only in the maintenance of health [25, 26] but also in the preventing of cardiovascular disease. Thus, Ahmet et al. [27] showed that a blueberry-enriched diet increased myocardial tolerance to ischemic damage in a rat model of postmyocardial infarction dilated cardiomyopathy.

Therefore, the objective of the current study was to assess the antioxidant properties and the effects against reperfusion injury in the isolated rat heart of a fermented nonalcoholic extract of Colombian blueberry, examining the role played by NO.

## 2. Materials and Methods

### 2.1. Plant Material

*Vaccinium meridionale* constitutes the raw material used for the fermentation in this research; berries fruits were harvested at the beginning of December 2009, in Colombia, zone “El Retiro” (2175 m.a.s.l) into Antioquia region. The plant and berries were identified by the Corporación Colombiana de Investigación Agropecuaria (Corpoica), La Selva, Colombia (voucher specimen number ILS 14050070). The berries were manually harvested at the commercially appropriate ripeness stage (fruit maturity was based on fruit surface color) and transported to the Laboratory of Food Science, Faculty of Sciences, National University of Colombia in hermetic plastic canisters. In this place, the berries were cleaned by removing leaves, stems, and unripe and damaged berries.

### 2.2. Blueberries Processing

The maceration was performed in the traditional fashion, punching the berries fruits (2 kg) several times at −18°C during 2 days. After prefermentative cold maceration, H_2_O purified (2 L) was added.

### 2.3. Fermentation Experiment

The berry juice was transferred into stainless steel tank (5 L, 320 × 144 × 2 mm), and the fermentation was carried out by the inoculation of 0.165 g/L of selected cultures of yeast *Saccharomyces cerevisiae* Meyen ex E.C. Hansen 1883 (ARS Culture Collection, USDA). The fermentation was performed at 25 ± 2°C for 10 days. The soluble solids were adjusted to 30 °Brix with sugar. The fermentation was stopped by the addition of 40 mg SO_2_/L. Then, the fermented berry product was decanted, treated with albumin (0.4 g/L), and filtered through filter plates (45 *μ*m).

### 2.4. Standard Chemical Analysis

The total titratable acidity was assessed by titration with sodium hydroxide (0.1 N) and expressed as tartaric acid. The pH value was measured using a digital pH meter (Hanna Instruments, Buenos Aires, Argentina). Total soluble solids (°Brix) were measured as Brix using a refractometer RX-5000 (Atago, Tokyo, Japan). The alcoholic title was quantified as ethanol by GC-FID, using an Agilent 6890N GC system (Ramsey, MN, USA). The values of these parameters at the beginning and at the end of fermentation process are showed in Table 1. HPLC analysis was carried out using a column Zorbax C18 (250 × 4.6 mm, 5 *μ*m particle size), oven temperature 38°C. The solvents used were acetonitrile/formic acid/water (3 : 10 : 87) as solvent A and acetonitrile/formic acid/water (50 : 10 : 40) as solvent B. The elution gradient was as follows: 0 min 94% A and 6% B; 10 min 80% A and 20% B; 20 min 60% A and 40% B; 30 min 50% A and 50% B; 35 min 94% A and 6% B. The flow rate was 0.8 mL/min. Detection was measured at 525 nm. All analyses were made in triplicate.

### 2.5. Preparation of the Nonalcoholic Extract (BE)

**Figure 1:**
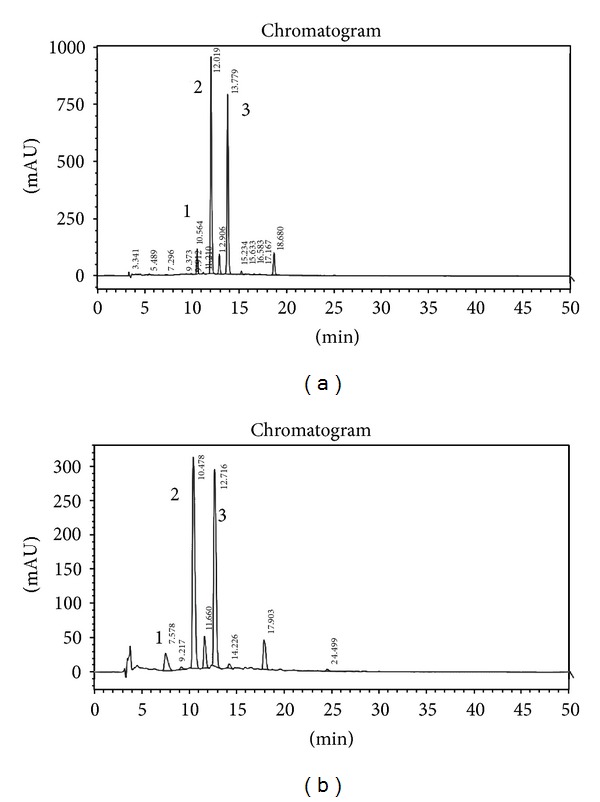
Anthocyanins profile. HPLC-UV chromatograms of fermented blueberry, (a) corresponds to the anthocyanins profile at the beginning of the fermentation process and (b) corresponds to the anthocyanins profile at the end of the fermentation process. Peak identification: 1, Delphinidin 3-glucoside; 2, cyanidin-3-glucoside; 3, petunidin-3-glucoside. Chromatographic analysis was carried out using a column Zorbax C18 and mixtures of acetonitrile/formic acid/water as mobile phase. The flow rate was 0.8 mL/min and detection was measured at 525 nm.

The extract of berries fermented (200 mL) was vacuum-evaporated (<30°C). The berry juice (BJ) was used as control. After being filtered, the aqueous extract was lyophilized and dry matter was maintained at −20°C until used. The nonalcoholic extract was chromatographed by HPLC and a representative chromatogram is included in Figure 1. The solid residue was 0.04 and 0.18 g/mL for BE and BJ, respectively. The residues were solubilized in deionized or Ringer's solution immediately before the assays.

### 2.6. Determination of Total Phenols and Total Anthocyanins Contents

Total phenolic content was measured by using the Folin-Ciocalteu. Results were expressed as caffeic acid equivalents/mg of dry weight of the extract [28]. Total anthocyanins were determined by using a pH differential method [29]. Results were expressed as mg of cyanidin-3-glucoside equivalents/mg of dry weight of the extract.

### 2.7. Total Antioxidant Activity

#### 2.7.1. 1,1-Diphenyl-2-picryl-hydrazyl (DPPH) Scavenging Activity

Reduction of the stable free radical DPPH was determined with the aid of a modified version of the method described by Cavin et al. [30]. The results are expressed in *μ*g caffeic acid equivalents/mg of dry weight of the extract.

#### 2.7.2. 2,2′-Azino-bis(3-ethylbenzothiazoline-6-sulphonic Acid (ABTS)^●^+^^ Scavenging Activity

Reduction of the stable ABTS radical cation was determined with the aid of a modified version of the method described by Re et al. [31]. Results are expressed as caffeic acid equivalents/mg of dry weight of the extract.

#### 2.7.3. Ferric Reducing Antioxidant Power (FRAP)

The FRAP assay was carried out to determine the reducing ability of the extracts with a method adapted from Benzie and Strain [32]. Results were expressed as *μ*mol of ascorbic acid equivalents/mg of dry weight of the extract.

### 2.8. Scavenging Activities

#### 2.8.1. Scavenging of Superoxide Anion (O_2_^∙−^)

O_2_^∙−^was generated through enzymatic oxidation of hypoxanthine with xanthine oxidase grade I (0.06 U) and was detected by the reduction of nitroblue-tetrazolium (NBT), monitored spectrophotometrically at 560 nm. Details of this assay have been described elsewhere [17]. Allopurinol was used as a reference.

#### 2.8.2. Scavenging of Peroxynitrite Anion (ONOO^−^)

ONOO^−^ was synthesized in a quenched flow reactor in accordance with the method described by Koppenol et al. [33]. The pyrogallol red bleaching assay was carried out as reported by Balavoine and Geletii [34].

#### 2.8.3. Nitration of Blood Serum Albumin (BSA) Induced by ONOO^−^

Reaction between ONOO^−^ and BSA was carried out as described by Jiao et al. [35]. Samples of control, BE, and BJ were analyzed by SDS-polyacrylamide gel electrophoresis (SDS-PAGE, 10%) following the Laemmli method by staining with Coomasie blue. The nitration of BSA was examined by the Western blot using an antinitrotyrosine polyclonal antibody (Cayman Chemicals, Ann Arbor, MI, USA). The bands were visualized with an ECL-Plus chemiluminescence detection system and analyzed by densitometric analysis using Scion Image software (Scion Co., Frederick, MD, USA).

#### 2.8.4. Hydroxyl Radical (OH^∙^) Scavenging Assay

The OH^∙^ scavenging assay was carried out according to the method described by Schinella et al. [17]. This assay was performed in triplicate with DMSO (20 mM) as a reference.

### 2.9. Isolated Heart Preparation

All procedures followed during this investigation conform to the Guide for the Care and Use of Laboratory Animals published by the US National Institutes of Health (NIH Publication No. 85–23, revised 1996) and to the guidelines laid down by the Animal Welfare Committee of La Plata School of Medicine. Male Wistar rats of 5-6 months of age were anesthetized with an intraperitoneal injection of sodium pentobarbital (60 mg/kg body wt). The heart was excised and perfused through the aorta by the nonrecirculating Langendorff technique with Ringer's solution (pH 7.4) and at 37°C. Heart rate was maintained at 280 ± 10 beats/min. A latex balloon was placed inside the left ventricle (LV) and connected to a Statham P23XL pressure transducer. The balloon was filled with water to provide an end-diastolic pressure (LVEDP) of 8–12 mmHg, and this volume was unchanged for the rest of the experiment. Coronary perfusion pressure was adjusted to approximately 60–70 mmHg and coronary flow was 11 ± 2 mL/min. Left ventricular pressure (LVP) was acquired by using an analog-to-digital converter and acquisition software (Chart V4.2.3 AD Instruments Sudamérica, Santiago de Chile, Chile).

#### 2.9.1. Experimental Protocols

After 10 min of stabilization, the following experimental protocols were performed. Ischemic control (IC) hearts (*n* = 6): hearts were subjected to 20 min of normothermic global ischemia followed by 30 min of reperfusion. Global ischemia was induced by stopping the perfusate inflow line and the heart was placed in a saline bath held at 37°C. Hearts treated with BE and BJ (*n* = 6 for each treatment): hearts were treated 10 min before ischemia and the initial 10 min of reperfusion with the blueberry extract (50 *μ*g/mL). The administration time was selected taking into account that reperfusion injury is the result of alterations that occur during ischemia and the first minutes of reperfusion [16]. Other hearts (*n* = 4) received 1 mM of N^G^-nitro-L-arginine methyl ester (L-NAME), a nonselective NOS inhibitor, 20 min before ischemia and during the reperfusion period. The L-NAME administration was extended through the entire reperfusion time to prevent the activation of the different NOS isoforms.

#### 2.9.2. Systolic and Diastolic Function

Myocardial contractility was assessed through the left ventricular developed pressure (LVDP), obtained by subtracting LVEDP to the LVP peak values and the maximal rise velocity of the left ventricular pressure (+*dP*/*dt*_max⁡_). Data were expressed as percentages of their respective preischemic values. The diastolic function was evaluated through the isovolumic LVEDP.

### 2.10. Biochemical Determinations from Isolated Heart Preparation

At the end of reperfusion, hearts were frozen in liquid N_2_ and kept at −70°C until the moment of assays.

#### 2.10.1. Assessment of Lipid Peroxidation

A portion of LV was homogenized in a solution composed by 25 mM KH_2_PO_4_-140 mM KCl at pH = 7.4 with a Polytron homogenizer. The samples were centrifuged and in the supernatant, an index of lipid peroxidation TBARS was determined by a spectroscopic technique and expressed as nmol MDA/g tissue weight [36].

#### 2.10.2. Reduced Glutathione (GSH)

Aliquots of homogenate were used to assess reduced glutathione content (GSH) according to Ellman's method [37] and expressed as nmol GSH/g tissue weight.

#### 2.10.3. Western Blot Analysis

Other portion of LV was homogenized in ice-cold RIPA buffer (300 mM sucrose, 1 mM DTT, 4 mM EGTA, 20 mM Tris pH 7.4, 1% Triton X, 10% protease cocktail, 25 *μ*M NaF, and 1 *μ*M orthovanadate), centrifuged at 10000 ×g for 15 min at 4°C. The supernatant was collected and subjected to SDS-PAGE. The samples were transferred to a PVDF membrane (2 h). Equal loading of samples was confirmed by Ponceau S staining. Membranes were blocked with 5% nonfat milk in Tris-buffered saline (pH 7.5) containing 0.1% Tween (TBS-T) and probed overnight at 4°C with antibodies anti-eNOS (Sigma-Aldrich, St. Louis, MO, USA), anti-Akt (Calbiochem, Merck Millipore Darmstadt, Germany). Membranes were washed four times for 10 min in TBS-T prior to the addition of anti-rabbit secondary antibody (1 : 1000 dilution) and the antibody-antigen complexes were developed using a chemiluminescent system (ECL Plus; GE Healthcare, Buckinghamshire, UK).

### 2.11. Statistical Analysis

Data were expressed as means ± SE. Statistical analysis was performed by one-way analysis of variance (ANOVA) followed by Student-Newman-Keuls multiple comparisons test. Differences were considered significant at *P* < 0.05.

## 3. Results

### 3.1. Antioxidant Activity

**Figure 2:**
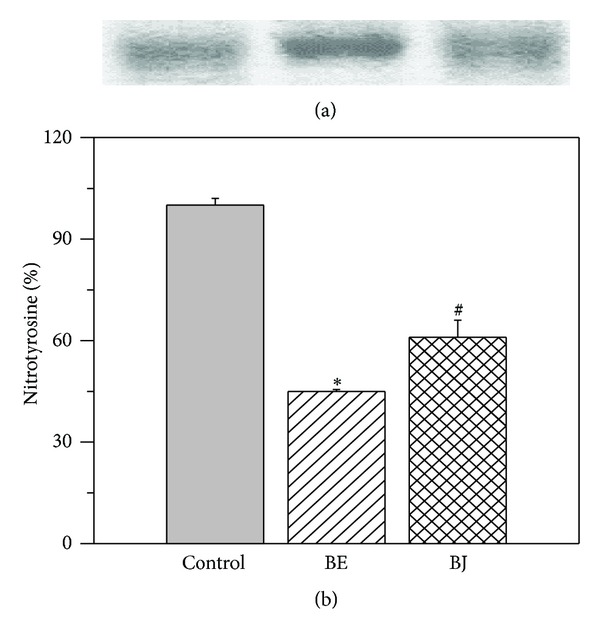
(a) Degradation; (b) nitration of BSA induced by ONOO^−^. Both extracts attenuated the degradation and nitration of BSA compared to control. However, the effects of fermented berry extract (BE) were significantly higher than that of berry juice (BJ). *P < 0.05 with respect to control; ^#^ *P* < 0.05 with respect to BE.

The total antioxidant activity was measured through the ability of the extracts to reduce DPPH^∙^  ABTS^∙^^+^ and Fe^3+^- and the content of total phenols and anthocyanins of BE was higher than BJ. BE also showed a higher O_2_^∙−^ and ONOO^−^ scavenging activity in comparison to BJ (Table 2). Moreover, it was detected as an attenuation of BSA degradation (Figure 2(a)) and nitration (Figure 2(b)) in the presence of BE. As indicated by graphic bar BE and BJ significantly decreased the percentage of nitrotyrosine compared to control (Figure 2(b)). However, the change produced by BJ was lesser to that obtained by BE. At a final concentration of 100 *μ*g/mL, none of the extracts were able to scavenge the OH^∙^ produced in the Fe^3+^-EDTA + H_2_O_2_ system in the presence or the absence of ascorbate. Moreover, the results showed a significant increase of deoxyribose degradation after the treatment with the two samples in the absence of ascorbate (data not shown).

### 3.2. Myocardial Systolic and Diastolic Function and Oxidative Damage of Cardiac Tissue

**Figure 3:**
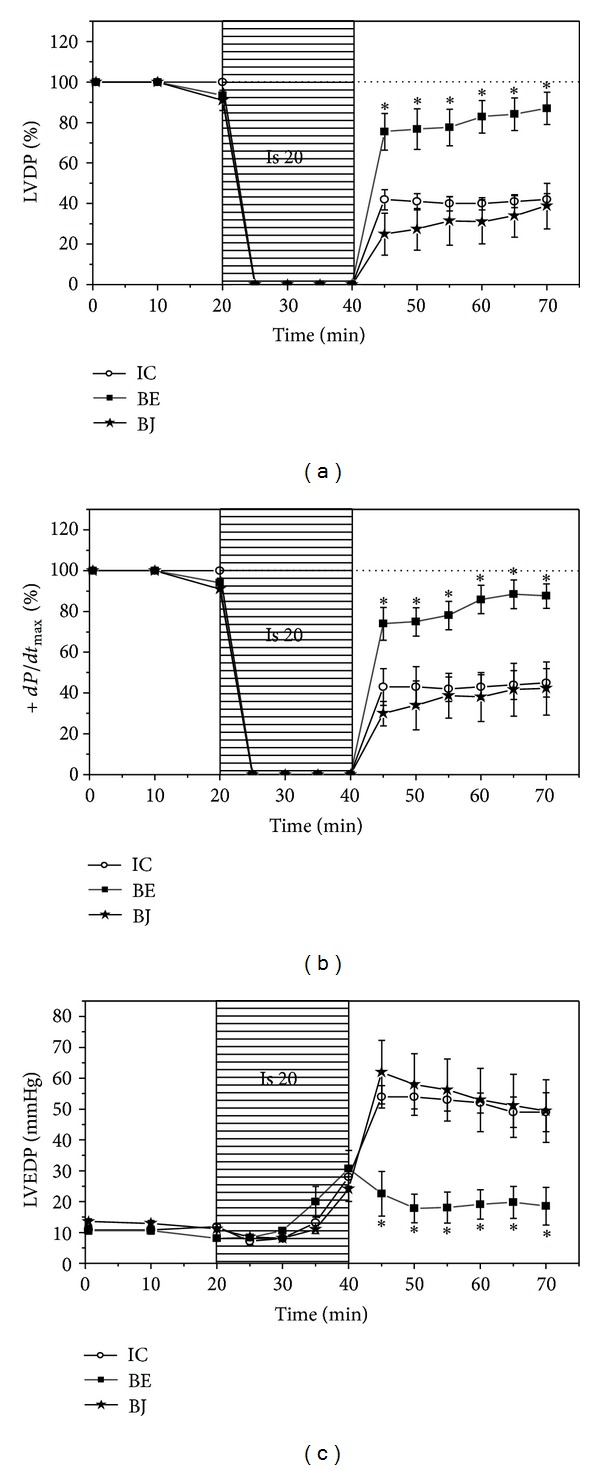
(a) Time course of left ventricular developed pressure (LVDP); (b) maximal rise velocity of left ventricular pressure (+*dP*/*dt*_max⁡_); left ventricular end-diastolic pressure (LVEDP) during ischemia and reperfusion in ischemic control hearts (IC, *n* = 6) and in hearts treated with the fermented berry extract (BE, *n* = 6) and berry juice (BJ, *n* = 6). Note that LVDP and +*dP*/*dt*_max⁡_ increased and LVEDP decreased during reperfusion only after treatment with BE. *P < 0.05 with respect to IC.

**Figure 4:**
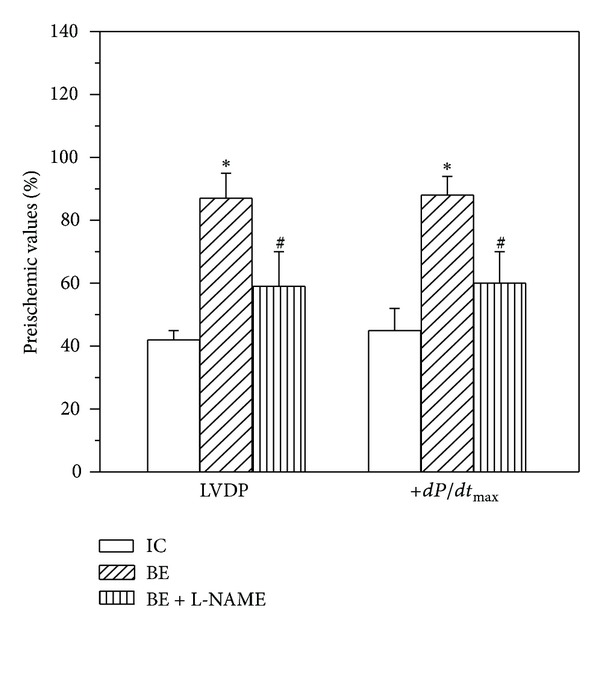
Values of left ventricular developed pressure (LVDP) and maximal rise velocity of left ventricular pressure (+*dP*/*dt*_max⁡_) at the end of reperfusion period in ischemic control hearts (IC, *n* = 6) and in hearts treated with fermented berry extract (BE, *n* = 6) and BE + L-NAME (NOS inhibitor) (*n* = 4). Note that L-NAME (NOS inhibitor) significantly attenuated the improvement of LVDP and +*dP*/*dt*_max⁡_ observed after BE treatment. *P < 0.05 with respect to IC; ^#^ *P* < 0.05 with respect to BE.

After BE treatment the postischemic recovery improved, reaching LVDP values significantly higher than that obtained in IC hearts (87 ± 8% versus 42 ± 3% at the end of reperfusion period). The treatment with BJ did not modify the postischemic recovery detected in IC hearts and at the end of reperfusion LVDP reached a value of 39 ± 9% (Figure 3(a)). A similar pattern was obtained when +*dP*/*dt*_max⁡_ was analyzed (Figure 3(b)). The ischemic contracture observed in IC hearts was not modified after all the treatments applied. The treatment with BE but not with BJ was able to attenuate the increase of LVEDP detected during reperfusion in IC hearts (18 ± 6 mmHg versus 49 ± 6 mmHg at the end of reperfusion period) (Figure 3(c)). The NOS inhibition with L-NAME abolished the protective effect of BE on postischemic recovery of systolic myocardial function (Figure 4). In addition, LVEDP was impaired after the combination of BE + L-NAME reaching a value of 59 ± 14 mmHg at the end of the reperfusion period.

**Figure 5:**
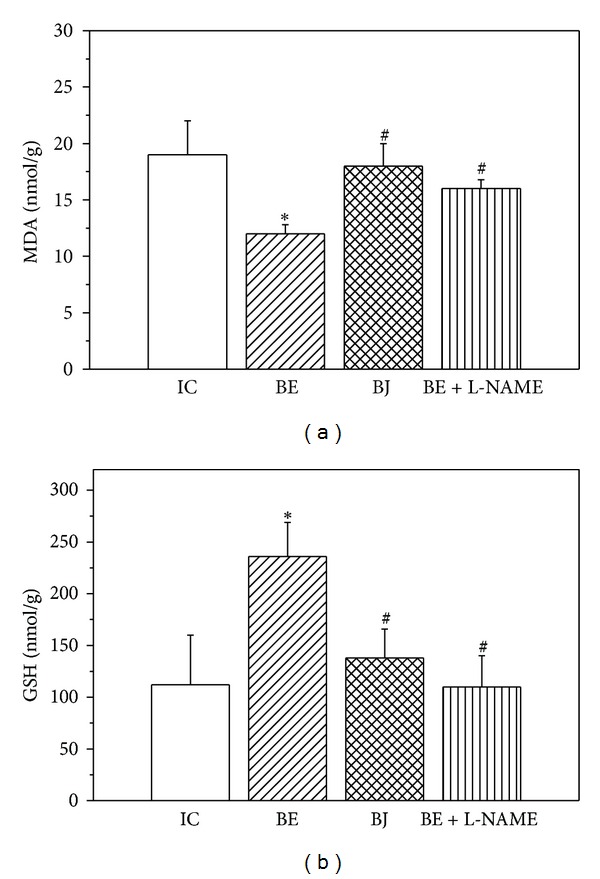
(a) Effects of fermented berry extract (BE, *n* = 6) and berry juice (BJ, *n* = 6) and BE + L-NAME (*n* = 4) on lipid peroxidation (MDA); (b) reduced glutathione (GSH). The treatment with BE attenuated lipid peroxidation and preserved GSH levels in comparison to IC hearts. These favorable changes were annulled when BE was administered in the presence of NOS inhibitor, L-NAME. *P < 0.05 with respect to IC; ^#^ *P* < 0.05 with respect to BE.

TBARS content (Figure 5(a)) diminished and GSH level (Figure 5(b)) was partially preserved when hearts were treated with BE but not after BJ compared with untreated hearts. These beneficial effects were annulled in presence of NOS inhibition detecting an increase of lipid peroxidation and less GSH level (Figures 5(a) and 5(b): BE + L-NAME column).

**Figure 6:**
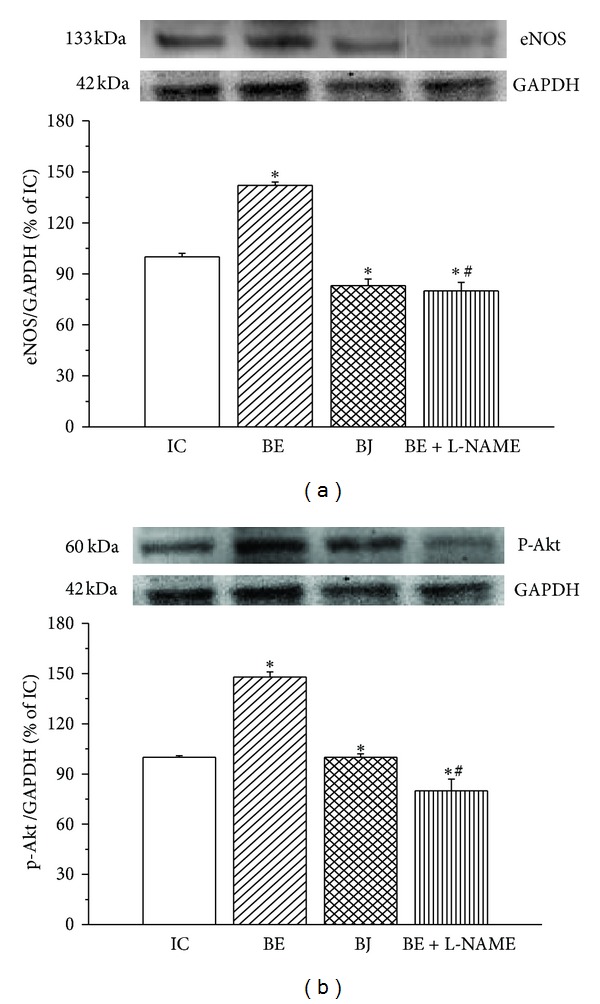
(a) Representative Western blots and mean data of eNOS; (b) phospho-Akt (p-Akt) protein expression normalized to GAPDH in ischemic control hearts (IC, *n* = 6) and in hearts treated with both fermented berry extract (BE, *n* = 6) and berry juice (BJ, *n* = 6) and BE + L-NAME (*n* = 4). It is observed that only BE increased the expression of eNOS and p-Akt and this increase was abolished when NOS was inhibited with L-NAME. *P < 0.05 with respect to IC; ^#^ *P* < 0.05 with respect to BE.

The treatment with BE increased the expression of eNOS compared to that detected in IC hearts while BJ treatment decreased it (Figure 6(a)). When BE was administered in the presence of NOS inhibitor L-NAME, eNOS expression decreased to levels significantly lesser than those of IC. The expression of p-Akt (Figure 6(b)) showed the same changes except in the BJ group in which the p-Akt expression was not different to IC hearts.

## 4. Discussion

The present study provides evidence about the antioxidant properties and cardioprotective effects of a nonalcoholic extract of fermented Colombian blueberry in isolated rat heart. This fermented extract showed higher antioxidant capacity (Figure 2 and Table 2) in comparison to BJ attributed to their higher content of total phenols and anthocyanins. It also detected a different composition of polyphenols (Figure 1) in BE in comparison to BJ.

In isolated rat hearts, the treatment with BE improved the postischemic recovery of systolic and diastolic myocardial function. Therefore, an increase of contractility parameters as left ventricular developed pressure (Figure 3(a)) and the maximal rise velocity of the left ventricular pressure (+*dP*/*dt*_max⁡_, Figure 3(b)) were detected in treated hearts when the coronary flow was restored. Simultaneously, a decrease of left ventricular end-diastolic pressure was observed during reperfusion in hearts treated with BE indicating that myocardial diastolic stiffness was minor in the presence of the extract (Figure 3(c)). The figure also shows that the treatment with BJ did not improve the postischemic recovery of myocardial function displaying the absence of beneficial effects in our experimental conditions.

Which are the mechanisms involved in the cardioprotective action of BE? The NO derived principally of the constitutive endothelial nitric oxidase synthase (eNOS) is able to exert beneficial or deleterious effects. Thus, NO interacts with proteins via S-nitrosylation at specific cysteine residues to alter their function [38, 39] which has an important meaning regarding the excitation-contraction (EC)-coupling-related process [38]. In the normal range of O_2_^∙−^, S-nitrosylation is accommodated; however, it is inhibited when O_2_^∙−^ is increased [40]. As noted, increased O_2_^∙−^ interacts with NO to form ONOO^−^, a reactive species that is capable of triggering an array of cytotoxic processes, including lipid peroxidation, protein oxidation, and nitration (altering EC coupling) [41, 42]. In our experiments, the eNOS inhibition abolished the cardioprotective effects afforded by BE. Thus, an impairment of systolic and diastolic myocardial function was evident in the presence of L-NAME (Figure 4) indicating that NO plays a crucial role in the attenuation of contractile dysfunction that follows the ischemia and the reperfusion.

On the other hand, it is well known that brief episodes of myocardial ischemia and reperfusion are associated with the generation of ROS [1–3, 43]. ROS cause lipid peroxidation first leading to reversible damage and eventually to necrosis and/or apoptosis. The administration of antioxidants or free radicals scavengers is able to limit the evolution of myocardial damage reducing ROS-induced lipid peroxidation [44]. In this study, we found that TBARS decreased and the level of GSH was higher in hearts treated with BE in comparison to ischemic control hearts displaying an attenuation of oxidative stress by the extract. However, lipid peroxidation increased and GSH levels decreased in hearts treated with L-NAME, showing the predominance of ROS and the consequent oxidative damage when eNOS was inhibited (Figures 5(a) and 5(b)). Western blot analysis revealed that eNOS expression of hearts treated with BE increased compared to untreated hearts and this effect was annulled by eNOS inhibition (Figure 6(a)). Which can be the pathway of eNOS activation by BE? As shown in Figure 6(b), the level of Akt phosphorylation also increased in hearts treated with BE, effect that was abolished by eNOS inhibition. Taking into account that phosphatidylinositol-3-kinase- (PI3K-) Akt can activate eNOS [45], our data suggest that Akt is involved in the activation of eNOS mediated by BE.

Because TBARS decrease coincides with higher eNOS expression level and that L-NAME treatment prevented these changes, it might at first be assumed that eNOS-derived NO could be involved in the attenuation of oxidative stress that occurs during ischemia and reperfusion. In fact, a close relationship between NO and oxidative stress has long been proposed [46]. Moreover, data from Clancy et al. [47] indicate that NO reacts with intracellular GSH forming S-nitrosoglutathione, compound which may function as stable intermediate of NO activity. Therefore, the improvement of GSH level added to the *in vitro* demonstration of the capacity of the extract to remove O_2_^●−^ and ONOO^−^ could be indicating the presence of a higher NO bioavailability in hearts treated with BE compared to untreated hearts. Therefore, these data are strong arguments to justify the involvement of NO in the attenuation of postischemic myocardial dysfunction afforded by the extract.

This study clearly demonstrates for the first time the protective effect of a nonalcoholic extract of berry fruit from *Vaccinium meridionale* against reperfusion injury in isolated rat heart. These cardioprotective properties may be linked to the ability of the extract to induce eNOS expression via Akt and to scavenge ROS. From these results, we propose that the “mortiño” extract could be a promising therapeutic approach against myocardial dysfunction. Moreover, being the fermented extract of berry fruit a source of polyphenols, it could be used as a functional food to prevent or mitigate pathologies associated to oxidative stress [48].

## Figures and Tables

**Table 1:** Physicochemical characteristics of berry process fermentation.

Days	1	10
Treatment	BE	BE
Ethanol %^(a)^	0.20	11.05
pH	2.58	2.82
°Brix	30	6.2
Total titratable acidity^(b)^	7.01	8.89

^(a)^Expressed as alcoholic title % w/v; ^(b)^Expressed as g/L of tartaric acid. BE: berry extract.

**Table 2:** Phenolics compounds, total antioxidant activity, and reactive oxygen species (ROS) scavenging properties of fermented berry extract (BE) and berry juice (BJ).

Sample	Phenolic compounds		Antioxidant activity			ROS scavenging	
	Total phenolics^(a)^	Total anthocyanins^(c)^	DPPH^(a)^	ABTS^•+(a)^	FRAP^(b)^	O_2_^•−(a)^	ONOO^−(b)^
BE	49	34	23	73	220	5.6	5.0
BJ	18	12	5	24	72	2.3	1.3

^(a)^Results are expressed as caffeic acid equivalent (*μ*g/mg dry weight of extract); ^(b)^results are expressed as ascorbic acid equivalent (*μ*mol/mg dry weight of extract); ^(c)^results are expressed as cyanidin-3-glucoside equivalents (*μ*mol/mg dry weight of extract).
